# Cost-effectiveness analysis of vaccinating children in Malawi with RTS,S vaccines in comparison with long-lasting insecticide-treated nets

**DOI:** 10.1186/1475-2875-13-66

**Published:** 2014-02-24

**Authors:** Mikyung Kelly Seo, Peter Baker, Karen Ngoc-Lan Ngo

**Affiliations:** 1 MSc in Health Policy, Planning and Financing, London School of Hygiene and Tropical Medicine and London School of Economics, London, UK; 2 MSc in International Health Policy, London School of Economics, London, UK

**Keywords:** RTS,S vaccine, Malaria vaccine, Cost-effectiveness analysis, Long-lasting insecticide-treated net, Bed net, Malawi, Malaria

## Abstract

**Background:**

New RTS,S malaria vaccines may soon be licensed, yet its cost-effectiveness is unknown. Before the widespread introduction of RTS,S vaccines, cost-effectiveness studies are needed to help inform governments in resource-poor settings about how best to prioritize between the new vaccine and existing malaria interventions.

**Methods:**

A Markov model simulated malaria progression in a hypothetical Malawian birth cohort. Parameters were based on published data. Three strategies were compared: no intervention, vaccination at one year, and long-lasting, insecticide-treated nets (LLINs) at birth. Both health service and societal perspectives were explored. Health outcomes were measured in disability-adjusted life years (DALYs) averted and costed in 2012 US$. Incremental cost-effectiveness ratios (ICERs) were calculated and extensive sensitivity analyses were conducted. Three times GDP per capita ($1,095) per DALY averted was used for a cost-effectiveness threshold, whilst one times GDP ($365) was considered ‘very cost-effective’.

**Results:**

From a societal perspective the vaccine strategy was dominant. It averted 0.11 more DALYs than LLINs and 0.372 more DALYs than the no intervention strategy per person, while costing $10.04 less than LLINs and $59.74 less than no intervention. From a health service perspective the vaccine’s ICER was $145.03 per DALY averted, and thus can be considered very cost-effective. The results were robust to changes in all variables except the vaccine and LLINs’ duration of efficacy. Vaccines remained cost-effective even at the lowest assumed efficacy levels of 49.6% (mild malaria) and 14.2% (severe malaria), and the highest price of $15. However, from a societal perspective, if the vaccine duration efficacy was set below 2.69 years or the LLIN duration of efficacy was greater than 4.24 years then LLINs became the more cost-effective strategy.

**Conclusion:**

The results showed that vaccinating Malawian children with RTS,S vaccines was very cost-effective from both a societal and a health service perspective. This result was robust to changes in most variables, including vaccine price and vaccine efficacy, but was sensitive to the duration of efficacy of the vaccine and LLINs. Given the best evidence currently available, vaccines can be considered as a very cost-effective component of Malawi’s future malaria control programmes. However, long-term follow-up studies on both interventions are needed.

## Background

Malaria is one of the leading causes of morbidity and mortality in Africa [1]. Malawi’s 14.9 million people live in a hyperendemic malarial area with stable and high malaria transmission rates, predominantly caused by *Plasmodium falciparum*[2,3]. Malawians suffered 6.1 million cases of malaria in 2009, accounting for 30% of outpatient appointments and costing $2.7million [4].

National malaria control programmes include a selection of measures such as environmental measures, improved case management, intermittent preventative treatment, indoor residual spraying, and insecticide-treated nets (ITNs). ITNs have been established as one of the most cost-effective interventions for controlling malaria, costing $4-10 per disability-adjusted life year (DALY) averted [5]. Long-lasting, insecticidal nets (LLINs) have replaced ITNs due to their superior cost-effectiveness and Malawi plans to increase the coverage of LLINs up to 80% by 2015 [6,7]. Although no malaria vaccine is currently licensed, the RTS,S vaccine against *P. falciparum* is considered to be the most promising. A phase III trial of the RTS,S vaccine is underway, and early results show comparable efficacy to LLINs [8-15].

Given the limited budget for health expenditures in Malawi, it may not be immediately affordable to add vaccination to Malawi’s current malaria control plan. As such, it is vital that the government and donors are able to determine which interventions to prioritize in their malaria control programme. Therefore, determining the cost-effectiveness of implementing a new vaccination programme in comparison to Malawi’s proposed widespread provision of LLINs is a key concern, as this will enable prioritization of scarce resources [16]. Existing research has not addressed this question adequately. The cost-effectiveness of a malaria vaccine in Tanzania has recently been estimated at between $12 and $190 per DALY averted, depending on the vaccine price [17,18]. This result provides limited insight however, since it did not compare vaccines directly to a best-alternative option, such as LLINs. Currently, only one study has directly compared the cost-effectiveness of vaccines and ITNs, but this was carried out in 1998, prior to the development of LLINs and the latest RTS,S trials [19]. Thus, the cost-effectiveness of the new vaccine has not yet been examined rigorously.

Furthermore, since the RTS,S vaccines are likely to be licensed in a couple of years, an early evaluation would help reduce any unnecessary lead time between product regulatory approval and its utilization [20]. Hence, this study examines the cost-effectiveness of a strategy to deliver RTS,S vaccines to one-year old children in Malawi compared with a strategy to provide LLINs to newborns and a no intervention strategy for malaria control in Malawi.

## Methods

### Decision model

A Markov model was constructed using the TreeAge Pro 2009 software and was used to compare three strategies for controlling malaria in Malawi: vaccines, LLINs and no intervention. A static model was chosen because herd immunity would be unachievable with the current scenario of vaccinating infants only, since adults would continue to be a large parasite reservoir [21,22].

**Figure 1:**
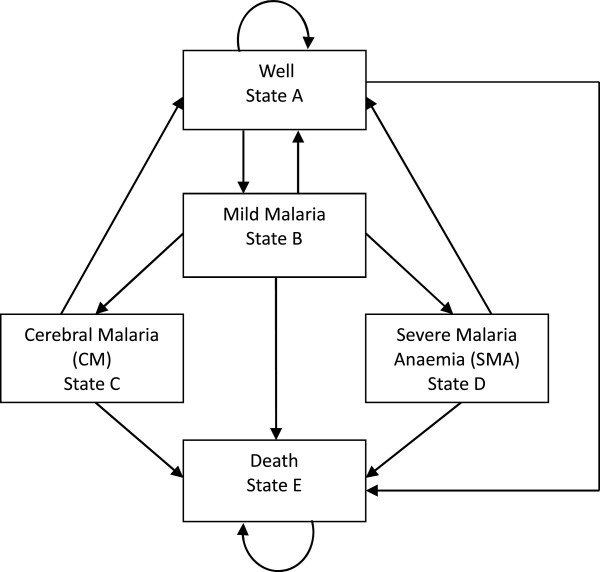
**Structure of the model.** Every five days, cohort members face the probability of moving to the next health state as depicted by arrows in the diagram.

The population in the model was a hypothetical Malawian birth cohort. The cohort experienced two epidemiological processes. Firstly, the age-specific non-malaria mortality rate was applied, based on World Health Organization (WHO) life tables [23]. Secondly, malaria morbidity and mortality was modelled using five mutually exclusive health states – well, mild malaria, cerebral malaria (CM), severe malarial anaemia (SMA), and death (Figure 1) [24]. Cycle lengths were set at five days because one cycle approximates the average mild disease episode, and two cycles approximates the average duration of mild malaria and progression to severe malaria (CM or SMA) [24]. Age-specific, annual epidemiological rates of each disease state were therefore transformed into five-day probabilities [25].

### Perspective

Health services and societal perspectives were employed for the data collection and the analysis. Its results were then reported separately.

### Transition probabilities

#### Comparator: providing no intervention

The transition probabilities for the no intervention strategy were derived from epidemiological data collected prior to the widespread provision of ITNs or LLINs. The best available estimates come from two meta-analyses of active case-finding studies in sub-Saharan Africa countries with similar climates [24,26]. For the LLIN strategy and the vaccine strategy, these transition probabilities were adjusted by the efficacy of the intervention and the proportion of the population successfully protected. All probabilities used in this model are given in the Table 1 and the detailed transition matrices can be seen in Additional file 1.

**Table 1:** Base-case values and ranges used in sensitivity analysis

**Description**	**Value**			**Reference**
	**Base-case estimate**	**Range for sensitivity analysis**		**(reference for range)**
		**Low**	**High**	
**Costs (2012 USD)**				
**Disease costs**				
*Medical costs*				
Mild malaria				
Public health systems	0.51	0.26	0.77	[27] (±50%)
Households	0.50	0.21	1.00	[28] (±50%)
Severe malaria (CM, SMA)				
Public health systems	12.76	6.38	19.14	[27] (±50%)
Households	0.50	0.21	1.00	[28] (±50%)
*Non-medical costs*				
*(Productivity costs)*				
Mild, CM, SMA				
Adults	2.52	1.26	3.78	[16,27] (±50%)
Children	1.11	0.56	1.67	[16,27] (±50%)
Death	4.76	2.38	7.14	[16] (±50%)
**Vaccine costs**				
Price per dose	7	1	15	[29,30] (Min, Max)
Administration per dose	0.37	0.19	0.57	[30] (±50%)
**Total costs for 3 doses**	22.11	3.57	46.71	
**LLINs costs**				
LLIN per two persons	3.16	2.23	5.23	[31,32] (Min, Max)
**DALY-related parameters**				
Life expectancy	47.1 years			[23]
Disability weights				[33]
Mild malaria	0.191	0.172	0.211	
CM	0.471	0.443	0.471	
SMA	0.471	0.191	0.471	
Death	1			
**Effectiveness parameters**				
**Vaccine protection efficacy**				
Mild Malaria	0.539	0.496	0.578	[12] (95% CI)
Severe Malaria (CM, SMA)	0.313	0.142	0.448	[12] (95% CI)
Death	0.2	0.15	0.25	[19] (±25%)
Duration of protection	45 months	11 months	10 years	[14]
Vaccine coverage	0.85	0.64	0.99	[34,35] (Min, Max)
**LLIN protection efficacy**				
Mild Malaria	0.5	0.43	0.62	[36] (Min, Max)
Severe Malaria (CM, SMA)	0.45	0.2	0.63	[36] (95% CI)
Death	0.17	0.1	0.24	[36] (95% CI)
Duration of protection	3 years	3 years	5 years	[37] (Min, Max)
LLIN coverage	80%	55.4%	80%	[7] (Baseline (2010) - Target (2015))
LLIN use	88%	87%	90%	[38] (95% CI)
Discount rate%	3%	0%	6%	[39]

### Comparator: delivering LLINs to children

The LLIN strategy modelled the delivery of one LLIN for every two children through existing channels (antenatal clinics and at birth), reaching a coverage rate of 80% as per the government’s national strategy [7]. LLINs were chosen as a comparator intervention because they are highly cost-effective and because they form such a large part of the government’s malaria control strategy [6,7]. An 88% correct usage rate for LLINs was taken from a large Malawian survey in 2009 [38]. A Cochrane meta-analysis found that LLINs had a protective efficacy of 50% against mild cases, 45% against severe and 17% against death, and last up to three years, according to WHO data [36,37].

### Intervention: vaccinating children with RTS,S vaccines

The vaccine strategy represents the delivery of three doses of the RTS,S vaccine to infants as per the current phase III trial [40]. The model included infants who were to be vaccinated through the Expanded Programme on Immunization (EPI) and receive their last dose at age one. Therefore, for the effectiveness of the vaccine, data from the ongoing phase III trials in African children were used, which showed a protective efficacy of 53.9% against mild cases and 31.3% against severe cases if given to children aged five to 17 months [12,15]. Severe malaria includes CM and SMA. However, the clinical trials were not designed to study impact on mortality rates, and so an estimate of 20% from a previous modelling study was used [19].

New vaccines tend to achieve a lower coverage rate than long-standing vaccines. Given that Malawi’s coverage rate in 2011 was greater than 85% for all of its eight EPI vaccines, the coverage of the RTS,S vaccine was conservatively assumed to be 85% [34]. Since follow-up data from the phase III vaccine study are only available for the first year, the duration of vaccine efficacy is not known. Previously however, the RTS,S/AS02 vaccine retained its efficacy for 45 months in trials, therefore this was taken as the most likely duration of vaccine efficacy [14]. No booster doses were assumed in the model after this duration because the efficacy of a booster dose is not currently known.

### Health outcomes (DALYs)

DALYs averted were the primary health outcome of interest in this analysis, calculated using Malawian life expectancy [23,41]. Disability weights were assigned according to the global burden of disease (0.21 for mild malaria, 0.47 for CM) [33]. SMA does not have an official value so CM’s value was used and this assumption was tested in the sensitivity analyses. DALYs were calculated without age-weighting and with a 3% discount rate, as this has become standard practice [42].

### Costs

Costs were collected from both a health services perspective and from a societal perspective. All costs were collected from published literature (Table 1). All costs were inflated to 2012 US dollars, and discounted at 3% per year [43,44]. For costs of treatment by the healthcare provider, the data on Malawi’s average recurrent cost per malaria episode were used [27].

Vaccine costs are composed of vaccine price and administration costs. The vaccine price was derived from existing EPI vaccines of developing countries since no price for the malaria vaccine exists. Especially, among all EPI vaccines, the recent vaccines’ introductory prices were employed as reference prices [29,30]. Therefore, the vaccine price was varied from $1 to $15 per dose, using $7 as the base case. The recurrent administration cost of the Malawian diphtheria, tetanus, pertussis (DTP) vaccine ($0.37 per dose) was used for administration costs of RTS,S vaccines [30]. No need for significant new capital investments were assumed because infants will be vaccinated through existing EPI facilities, which already achieve over 85% vaccination coverage [34].

For LLIN costs, it used a detailed assessment of a Malawian ITN programme to estimate the average administration cost per ITN delivered and then added LLINs costs ($4.50) [31,32]. One LLIN is provided for every two children so the final cost was halved.

For the societal perspective, the model included the additional costs incurred by patients and carers due to illness. For patients’ out-of-pocket payments, it used data from a large 2009-10 Malawian household survey [28]. Then the human capital approach was adopted to estimate productivity losses due to illness. Wages lost due to adult illness and carers’ wages lost due to childhood malaria were calculated, using GDP per capita of Malawi and survey data on time lost due to malaria in Malawi [16,27]. Similarly, the lost productivity due to death in working-age (14-65) people was estimated from GDP per capita.

### Cost-effectiveness threshold (CET)

Interventions that are both cheaper and more effective than the alternatives are clearly the most cost-effective option and are said to be ‘dominant’. Interventions that are more expensive and more effective were only recommended as cost-effective if their incremental cost-effectiveness ratio (ICER) was below Malawi’s cost-effectiveness threshold (CET). It used the thresholds established by the 2003 World Health Report, which considered interventions with an incremental cost per DALY averted of less than three times a country’s GDP per capita ($1,095) ‘cost-effective’ and less than one times GDP ($365) as ‘very cost-effective’ [39].

### Sensitivity analysis

One-way sensitivity analyses were performed on all variables to assess their impact on the cost-effectiveness result using a tornado diagram. Table 1 shows the upper and lower limits used for each variable. If changes in the variable resulted in a change in the cost-effectiveness result, then a threshold analysis was carried out. Further analysis was carried out on vaccine price, efficacy and duration of efficacy because these parameters are particularly uncertain and a better understanding of how much they impact on the vaccine’s cost-effectiveness will inform future vaccine research and guide how the vaccine is incorporated into future malaria control programmes.

## Results

### Cost-effectiveness

In the baseline analysis (Table 2), the vaccine’s ICER shows that vaccinating infants with the RTS,S vaccine is very cost-effective for Malawi. When a societal perspective was taken, the vaccine strategy was the cheapest and the most effective intervention, dominating the other two strategies. Per person, it averted 0.11 more DALYs than LLINs and 0.372 more DALYs than the no intervention strategy, whilst costing $10.04 less than LLINs and $59.74 less than no intervention. When a health services perspective was taken, the vaccine’s ICER was $145.03 per DALY averted. This ICER is far below the CET of Malawi ($1,095). Because it is also below the GDP of Malawi ($365), according to WHO-CHOICE, this ICER can therefore be considered very cost-effective [39].

**Table 2:** Base-case scenario cost-effectiveness result from a health services’ and a societal perspective

**Perspective**	**Societal perspective**			**Health services perspective**		
**Strategy ranking**	**Vaccine**	**LLIN**	**No intervention**	**No intervention**	**LLIN**	**Vaccine**
Cost	$1,148.81	$1,158.81	$1,208.51	$7.69	$8.67	$24.68
Incremental cost		$10.03	$59.73		$0.97	$16.01
DALYs	4.241	4.351	4.613	4.613	4.351	4.241
DALYs averted		-0.11	-0.372		0.262	0.11
Cost/DALY	$270.89	$266.33	$262.00	$1.67	$1.99	$5.82
Incremental cost/DALY averted (ICER)		(Dominated)	(Dominated)		$3.72	$145.03

### Sensitivity analysis

#### Tornado diagram

One-way sensitivity analyses were performed to determine the impact of each variable on the model, whilst holding all other variables at their baseline values. These are depicted in tornado diagrams in Additional files 2 and 3. The net benefit produced by each strategy was sensitive to changes in discount rates, productivity losses, vaccine efficacy duration, vaccine coverage, vaccine efficacy, vaccine price, and LLINs efficacy duration. However, only the LLIN duration of efficacy, the vaccine duration of efficacy and the vaccine coverage rate had any impact on the cost-effectiveness result. To explore this further a threshold analysis was carried out on these three variables.

### Threshold analysis

The baseline analysis assumed that the RTS,S vaccine would be efficacious for 3.75 years and showed that it is cost-effective compared with LLINs. However, the threshold analysis from the societal perspective showed that vaccines were very cost-effective (i e, had an ICER under $365) only if the duration of vaccine efficacy was over 2.84 years (see Table 3). They were cost-effective (i e, had an ICER under $1,095) only if their duration was above 2.69 years. In other words, if vaccine duration fell below 2.69 years, LLINs appeared to be more cost-effective than vaccination. A similar result was observed from the health service perspective (Table 3).

**Table 3:** Threshold analysis results on the duration of vaccine efficacy

**Perspective**	**Societal**	**Health services**
Vaccine duration above which vaccines become cost-effective (CET = $1,090)	2.69 years	2.73 years
Vaccine duration above which vaccines become very cost-effective (CET = $365)	2.84 years	3.05 years

Likewise, three years was assumed to be the duration of LLINs’ efficacy in the baseline analysis. However, the threshold analysis from a societal perspective showed that vaccines were very cost-effective (i e, had an ICER under $365) only if the duration of LLIN efficacy was under 4.07 years (Table 4). They were cost-effective (i e, had an ICER under $1,090) only if their duration was under 4.24 years. In other words, if LLINs were effective longer than 4.24 years, vaccines appeared to be cost-ineffective compared to LLINs. A similar result was seen from the health service perspective (Table 4).

**Table 4:** Threshold analysis results on the duration of LLIN efficacy

**Perspective**	**Societal**	**Health services**
LLIN duration below which vaccines become cost-effective (CET = $1090)	4.24 years	4.18 years
LLIN duration below which vaccines become very cost-effective (CET = $365)	4.07 years	3.80 years

The baseline analysis assumed that the vaccine would cover 85% of the population. From a health service perspective, varying this from 64 to 99% did not affect the cost-effectiveness result. From a societal perspective the vaccine was always cost-effective (i e, ICER under $1,095), however the vaccine coverage rate had to be over 67.62% to be very cost-effective (i e, ICER under $365).

### Uncertainty in the vaccine price

Further analysis was carried out on the impact of changes of vaccine price to ICER because RTS,S vaccine price is basically unknown and is of critical interest to all stakeholders including multilateral donor agencies. Results are presented in the Table 5 and suggest that the vaccine strategy is very cost-effective through a range of prices.

**Table 5:** Changes in the vaccine’s ICER according to different vaccine price scenarios

**Vaccine price per dose**	**Societal perspective**		**Health services’ perspective**	
	**Strategy ranking**	**Incremental cost/DALY averted (ICER)**	**Strategy ranking**	**Incremental cost/DALY averted (ICER)**
**$1**	Vaccines		No intervention	
	LLINs	(Dominated)	LLINs	$3.72
	No intervention	(Dominated)	Vaccines	$6.46
**$3**	Vaccines		No intervention	
	LLINs	(Dominated)	LLINs	$3.72
	No intervention	(Dominated)	Vaccines	$52.65
**$5**	Vaccines		No intervention	
	LLINs	(Dominated)	LLINs	$3.72
	No intervention	(Dominated)	Vaccines	$98.84
**$7**	Vaccines		No intervention	
	LLINs	(Dominated)	LLINs	$3.72
	No intervention	(Dominated)	Vaccines	$145.03
**$9**	Vaccines		No intervention	
	LLINs	(Dominated)	LLINs	$3.72
	No intervention	(Dominated)	Vaccines	$192.22
**$11**	LLINs		No intervention	
	Vaccines	$1.46	LLINs	$3.72
	No intervention	(Dominated)	Vaccines	$237.41
**$13**	LLINs		No intervention	
	Vaccines	$47.65	LLINs	$3.72
	No intervention	(Dominated)	Vaccines	$283.60
**$15**	LLINs		No intervention	
	Vaccines	$93.84	LLINs	$3.72
	No intervention	(Dominated)	Vaccines	$329.78

From a societal perspective, both the LLINs and the no intervention strategies were dominated by the vaccine strategy until the vaccine price reached $9 per dose. However, the no intervention strategy was always dominated regardless of the vaccine price. The vaccine’s ICER at the top price of $15 per dose was $93.84 per DALY averted which can be considered very cost-effective for Malawi (i e, under $365 per DALY averted). From a health services perspective, the vaccine’s ICER gradually increased as the vaccine price was inflated, reaching $329.78 per DALY averted at the top price of $15 per dose, thus remaining very cost-effective for Malawi.

### Methodological uncertainty in the time horizon and discount rates

The model results remained consistent even when time horizons were varied from 20 to 60 years (see Additional file 4). Whilst the vaccine’s ICER was sensitive to changes in discount rates, the overall cost-effectiveness result did not change (see Additional file 5). From a societal perspective, the vaccine strategy was dominant over the other two options when discount rates up to 5% were applied. However, when 6% discount rate was used, vaccination was no longer dominant and an ICER of $50.53 per DALY averted was seen. From a health services’ perspective, the ICER of the vaccine strategy was $73.69 per DALY averted with a 0% discount rate, and $255.01 per DALY averted with a 6% discount rate. In other words, the RTS,S vaccine was found to be cost-effective regardless of changes in time horizons and discount rates.

### Two-way sensitivity analysis on the duration of vaccine efficacy and price

**Figure 2:**
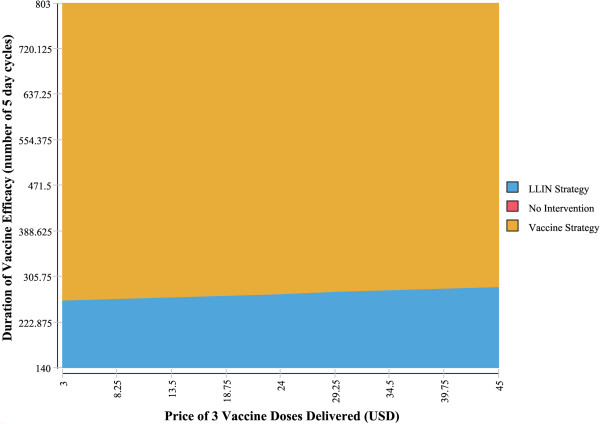
**Two-way sensitivity analysis of vaccine price and efficacy duration: health services’ perspective.** This graph demonstrates the range of vaccine prices and vaccine duration of efficacy where vaccines are more cost-effective than LLINs.

**Figure 3:**
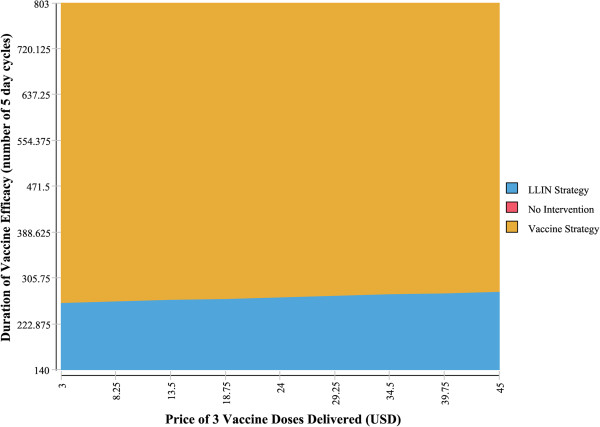
**Two-way sensitivity analysis of vaccine price and efficacy duration: societal perspective.** This graph demonstrates the range of vaccine prices and vaccine duration of efficacy where vaccines are more cost-effective than LLINs.

Two-way sensitivity analyses were carried out on the duration of vaccine efficacy and the vaccine price. As shown in Figures 2 and 3, vaccine price has little impact on which intervention the model finds to be the most cost-effective, however the impact of the duration of vaccine efficacy appears to be significant. This is in line with the threshold analysis results. In other words, the cost-effectiveness of RTS,S vaccines might be greatly determined by their duration of protection, but not by their price.

### Two-way sensitivity analysis on the vaccine efficacy and vaccine price

Even at the lowest assumed vaccine efficacy level and the highest price of $15 per dose, the two-way sensitivity analysis revealed that the vaccine strategy remained the most cost-effective intervention for Malawi. The vaccine’s ICER was $614.62 per DALY averted from the health service perspective and $351.38 from the societal perspective.

## Discussion

The cost-effectiveness of an RTS,S malaria vaccination strategy was compared with two alternative strategies (no intervention and the provision of LLINs), using a Markov model. From a health services’ perspective, the vaccine’s base-case ICER was $145.03 per DALY averted, and this was less than Malawi’s very cost-effective threshold of US$365. From a societal perspective, the vaccine strategy was dominant (i e, it was cheaper and more effective than the two alternative strategies). Thus, this analysis showed that, compared to LLINs, delivering the RTS, S vaccine to one-year old children is very cost-effective for Malawi.

This is the first study to directly compare the cost-effectiveness of the RTS,S vaccine with LLINs. Previous cost-effectiveness studies on the malaria vaccine support the finding that vaccination is cost-effective compared with respective comparator interventions. In 2006, Tediosi and colleagues compared a combined programme of vaccination plus malaria case management with only malaria case management in Tanzania from a societal perspective [17]. They evaluated the cost-effectiveness of vaccination depending on different vaccine price scenarios. When the lowest price of $1 per dose was assumed, the ICER was $8 per DALY averted. When the highest price range was assumed ($20 per dose), the vaccine’s ICER was $140 per DALY averted. It is in line with the findings from a societal perspective that the vaccine strategy dominated LLINs at the price of $1 per dose and produced an ICER of $93.84 per DALY averted when the highest vaccine price was assumed to be $15 per dose.

Further support for the results of this study comes from a recent modelling paper on different malaria vaccine types, undertaken from a health services’ perspective, showed that the vaccine’s ICER was $160 per DALY averted at a price of $10 per dose [18]. This is similar to the findings from a health services’ perspective that the vaccine’s ICER was $52.65 at a price of $3 per dose and $329.78 at $15 a dose. These previous findings are not perfectly comparable to this study because they lack a highly cost-effective comparator, such as LLINs. Nevertheless, they support the conclusion that the RTS,S vaccines are cost-effective over a wide range of different vaccine price scenarios.

Graves’ study [19] appears to be the most comparable study to the analysis of this study since he used ITNs as a comparator. It found that vaccination was cheaper and less effective than ITNs. This result is likely to be because the study was carried out before the development of the more efficacious RTS,S/AS01 vaccines. Furthermore, the study is unable to inform about the vaccine’s relative cost-effectiveness since ICERs were not calculated.

The result of this study was robust to changes in most variables. The RTS,S vaccine price is highly uncertain, and its efficacy has only been established in one trial. Therefore it is reassuring that the two-way sensitivity analysis found that even when the lowest vaccine efficacy value and the highest vaccine price were combined, the vaccine strategy was still very cost-effective. This is similar to previous findings that a malaria vaccine with 50% efficacy would be highly cost-effective [45].

If new malaria vaccines were protective for a lifetime with high efficacy, policy-makers’ decision making would be easier. The reality is, however, that any malaria vaccine seems unlikely to be highly efficacious in the near future [46]. Indeed, RTS,S vaccines are considered ‘leaky’, which means they may not protect individuals consistently from infection. Furthermore, Bejon *et al*. pooled vaccine efficacy data from phase II trials of 11 sites in Africa and analysed the variation in vaccine efficacy [47]. Their findings reported that vaccine efficacy is significantly varied by the duration of protection. For example, its efficacy declined to 0% at the three-year time of vaccination from 36% at the time of vaccination.

Likewise, this study result was particularly sensitive to the duration of vaccine efficacy and the duration of LLIN efficacy. The findings show that, in order to be cost-effective for Malawi, RTS,S vaccines must remain efficacious for longer than 2.69 years from a societal perspective (2.73 years from the health service perspective). This is in line with Engers and Godal’s analysis that the vaccine would need to provide three years of immunity with at least 30% efficacy [48]. Similarly, if LLINs’ duration of efficacy was set at greater than 4.24 years (from a societal perspective, 4.18 years from a health service perspective), then LLINs became the more cost-effective strategy. Thus, long-term follow-up studies of the vaccine’s phase III trial are recommended in order to produce more precise estimates of the vaccine’s efficacy and duration of protection, and therefore cost-effectiveness.

There are, however, several limitations in this study. First, although a static Markov model was justified due to a minimal or negligible effect of herd immunity in this study, a dynamic model might provide more information by capturing all indirect effects. However, if herd immunity is included, the cost-effectiveness through added benefits accrued on non-vaccinated people would be improved. This study therefore assumed a conservative stance and did not include the potential of herd immunity in this study. Second, a Markov model assumes the same transition probability for all participants irrespective of their previous health state, i e, it is memory-less [49]. Third, data sources of this study were all secondary and mostly lacked distributional information. Therefore, to avoid making excessive assumptions about data, probabilistic sensitivity analyses were not performed. Fourth, the model does not allow for any heterogeneity in the population. However, this is not a major concern in Malawi, which has high and consistent malarial transmission throughout the country [50]. Further studies in more heterogeneous countries may need to model geographical differences. Fifth, whilst this study modelled all malaria mortality and the vast majority of malarial morbidity, other malarial disease states, such as respiratory distress, were not explicitly modelled. However, this will not influence the result of the study significantly, since the vast majority of the DALYs accrued are due to mortality rather than morbidity. Sixth, in practice most governments in the future are likely to adopt a blend of interventions in their malaria control programmes, including both vaccines and LLINs, whereas this study modelled a strategy of vaccination against LLINs alone. It is because comparing the two interventions head-to-head enables governments and donors to prioritize scarce resources towards the more cost-effective components of a malaria control programme. Lastly, as more data from RTS,S vaccine phase III trials become available, more precise vaccine estimates, including the duration of vaccine efficacy and the efficacy of booster dose, the efficacy against malaria mortality should become known. This will enable the cost-effectiveness of RTS,S vaccines to be evaluated more precisely.

## Conclusion

This study found that a malaria vaccine strategy was very cost-effective for Malawi. From a health service perspective, although it was more expensive, it was more effective than LLINs or no intervention, and its ICER was far below the CET for Malawi. From a societal perspective it was cheaper and more effective, dominating the other two options. Although the vaccine strategy’s ICER was sensitive to a number of assumptions, including vaccine price, vaccine efficacy, duration of vaccine efficacy, discount rates for both outcomes and cost, the vaccine remained consistently ‘very cost-effective’ for Malawi. Therefore, the findings of this study should encourage the Malawian government to incorporate the vaccine into its control programme once it is licensed.

Although this study found vaccines to be more cost-effective than LLINs, in reality all malaria control programmes would be a blend of several interventions. Further studies are thus needed to explore the interaction between interventions. This will help guide decision makers on how best to incorporate the vaccine as part of an optimal package of interventions. Finally, further studies into the duration of efficacy of the vaccines would improve the understanding of their cost-effectiveness, which would be beneficial for policy makers.

## Abbreviations

CET: Cost-effectiveness thresholds; CM: Cerebral malaria; DALY: Disability-adjusted life year; DTP: Diphtheria, tetanus, pertussis; GDP: Gross domestic product; ICER: Incremental cost-effectiveness ratio; LLIN: Long-lasting insecticide-treated net; SMA: Severe malarial anaemia; WHO: World health organization.

## Competing interests

The authors declared that they have no competing interests.

## Authors’ contributions

MKS and PB developed the core concepts of the project, designed the model, analysed the data and findings, and wrote the manuscript. MKS, PB and KNLN collected data and edited the manuscript. All authors read and approved the final manuscript.

## Authors’ information

Mikyung Kelly Seo and Peter Baker are joint first authors. This research was done while the authors (MKS, PB) were students at LSHTM and LSE, and KNLN were students at LSE.

## Conflict of Interest

The research was done while MKS and PB were students at London School of Hygiene and Tropical Medicine (LSHTM) and London School of Economics (LSE), and KN was a student at LSE. We conducted this research driven by our own interests without funding received and with no third party involved. A majority part of the writing up of the paper was done while authors were students at these institutions. However, some part of the writing and editing was done while MKS and KN were working at PRMA Consulting and PB was at Imperial College London.

## Supplementary Material

Additional file 1: Table Transition matrix of cohort members under one year of age in each intervention. Click here for file

Additional file 2 Health Service Perspective Tornado diagram. Click here for file

Additional file 3 Societal Perspective Tornado diagram. Click here for file

Additional file 4: Table Changes of ICER according to different time-horizon scenarios. Click here for file

Additional file 5: Table Changes in vaccine’s ICER according to different discount rates. Click here for file
