# Supplementary material for: Cost-effectiveness analysis of vaccinating children in Malawi with RTS,S vaccines in comparison with long-lasting insecticide-treated nets
Source: Malar J. 2014 Feb 24;13:66. doi: 10.1186/1475-2875-13-66 (PMC4016032; doi:10.1186/1475-2875-13-66)
Supplement: Additional file 1: Table — Transition matrix of cohort members under one year of age in each intervention. [file 1475-2875-13-66-S1.docx]

| ***Additional File 1. Transition matrix of cohort members under 1 year of age in each intervention*** | | | | | |
| --- | --- | --- | --- | --- | --- |
| **'No Intervention' Arm** | | | | | |
|  | well | mild | CM | SMA | death |
| well | 0.97982 | 0.01932 | 0.00000 | 0.00000 | 0.00086 |
| mild | 0.96944 | 0.00000 | 0.00433 | 0.02304 | 0.00319 |
| CM | 0.80714 | 0.00000 | 0.00000 | 0.00000 | 0.19286 |
| SMA | 0.85714 | 0.00000 | 0.00000 | 0.00000 | 0.14286 |
| death | 0.00000 | 0.00000 | 0.00000 | 0.00000 | 1.00000 |
|  |  |  |  |  |  |
| **'Vaccines' Arm** | | | | | |
|  | well | mild | CM | SMA | death |
| well | 0.99099 | 0.00815 | 0.00000 | 0.00000 | 0.00086 |
| mild | 0.98229 | 0.00000 | 0.00239 | 0.01272 | 0.00261 |
| CM | 0.85514 | 0.00000 | 0.00000 | 0.00000 | 0.14486 |
| SMA | 0.89264 | 0.00000 | 0.00000 | 0.00000 | 0.10736 |
| death | 0.00000 | 0.00000 | 0.00000 | 0.00000 | 1.00000 |
|  |  |  |  |  |  |
| **'LLINs' Arm** | | | | | |
|  | well | mild | CM | SMA | death |
| well | 0.99180 | 0.00734 | 0.00000 | 0.00000 | 0.00086 |
| mild | 0.98724 | 0.00000 | 0.00160 | 0.00853 | 0.00263 |
| CM | 0.85322 | 0.00000 | 0.00000 | 0.00000 | 0.14678 |
| SMA | 0.89122 | 0.00000 | 0.00000 | 0.00000 | 0.10878 |
| death | 0.00000 | 0.00000 | 0.00000 | 0.00000 | 1.00000 |
| **Note: Our model is age-specific and thus, for simplicity, the first year of transition matrix is presented as an example.* | | | | | |
