# Supplementary figures and images for: Cost-effectiveness analysis of vaccinating children in Malawi with RTS,S vaccines in comparison with long-lasting insecticide-treated nets
Source: Malar J. 2014 Feb 24;13:66. doi: 10.1186/1475-2875-13-66 (PMC4016032; doi:10.1186/1475-2875-13-66)

# Health Service Perspective Tornado Analysis (Net Benefits)

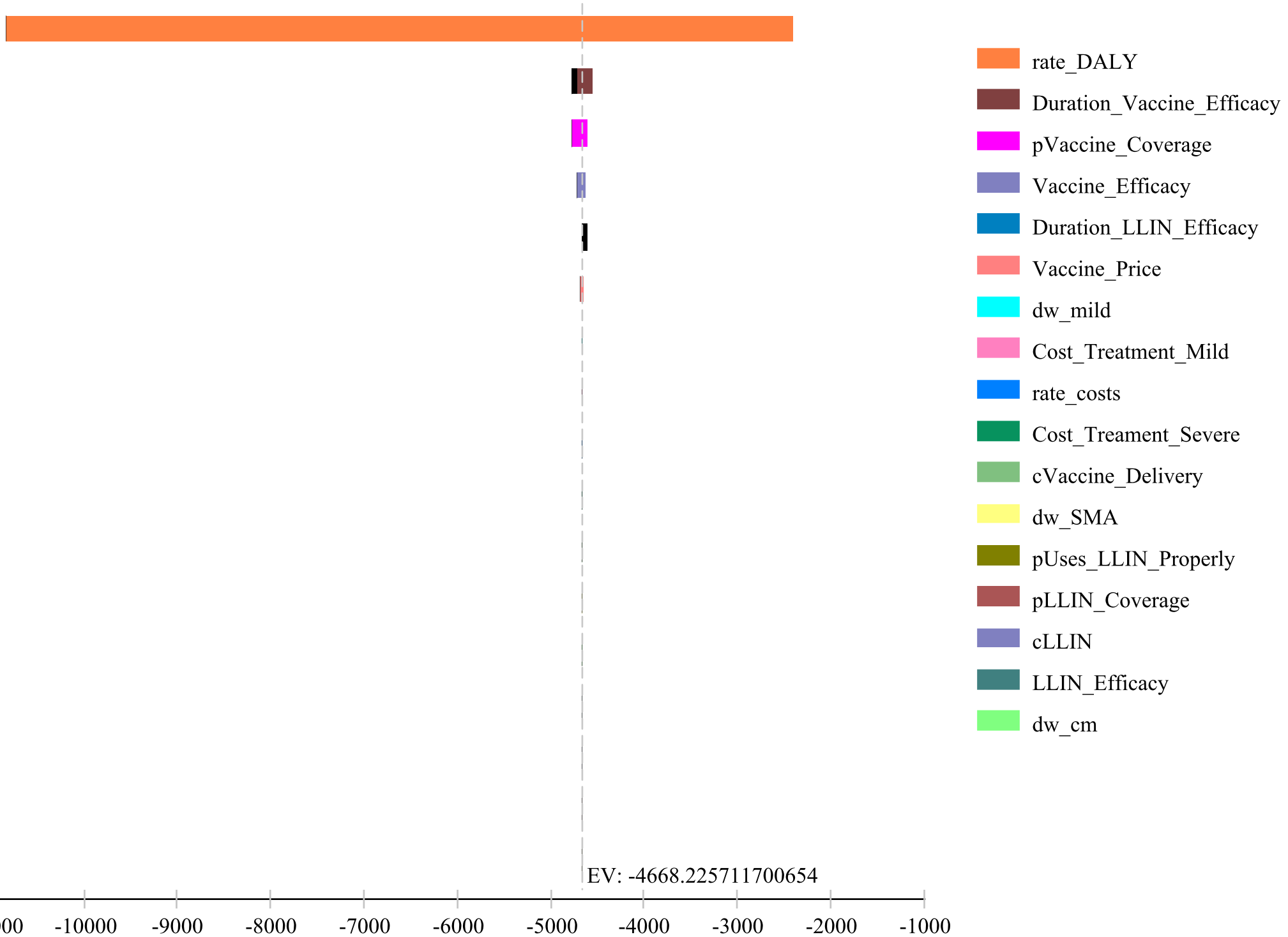

Supplement: Additional file 2 — Health Service Perspective Tornado diagram. [file 1475-2875-13-66-S2.pdf]

# Societal Perspective Tornado Analysis (Net Benefits)

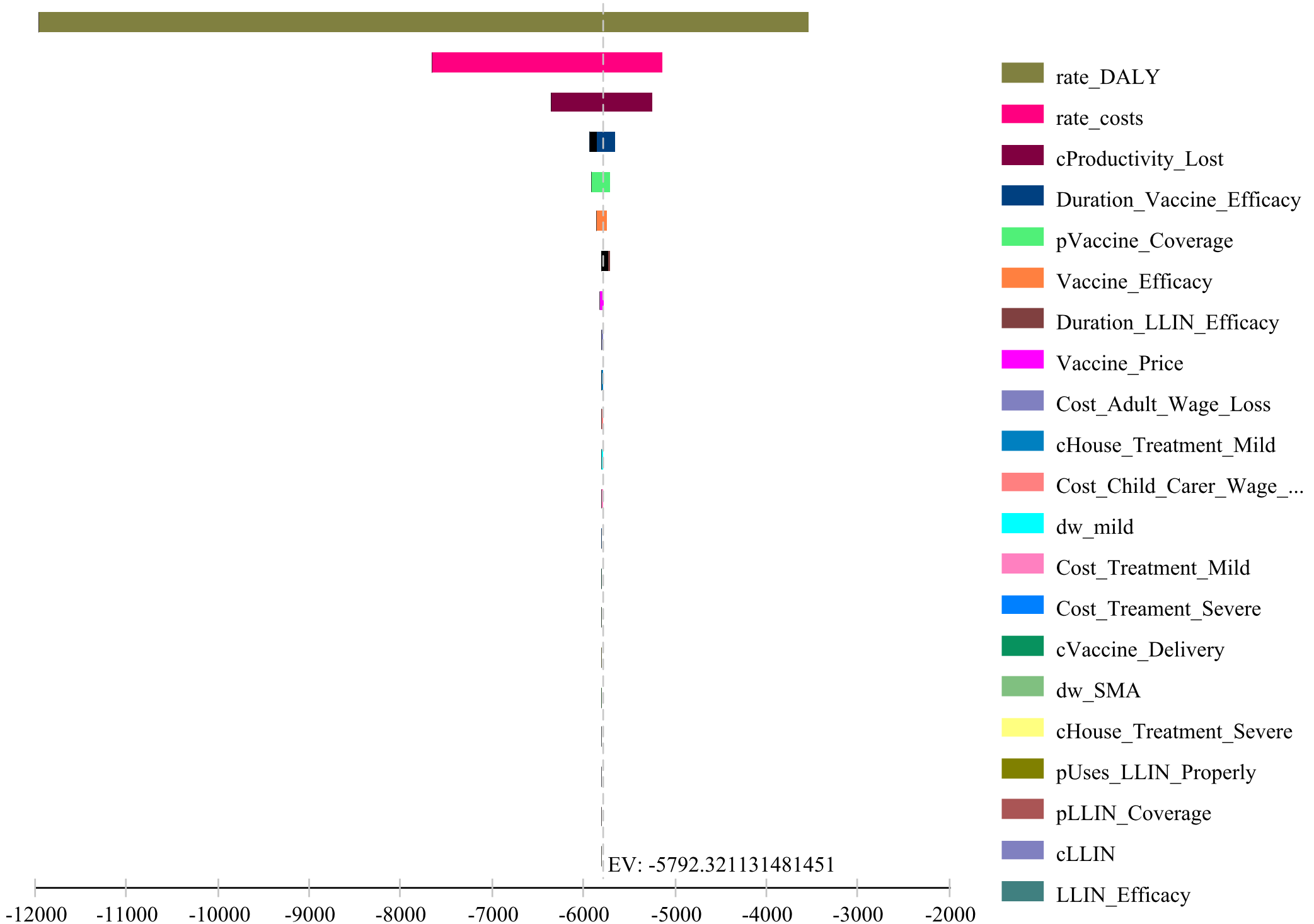

Supplement: Additional file 3 — Societal Perspective Tornado diagram. [file 1475-2875-13-66-S3.pdf]
