# Supplementary material for: Cost-effectiveness analysis of vaccinating children in Malawi with RTS,S vaccines in comparison with long-lasting insecticide-treated nets
Source: Malar J. 2014 Feb 24;13:66. doi: 10.1186/1475-2875-13-66 (PMC4016032; doi:10.1186/1475-2875-13-66)
Supplement: Additional file 4: Table — Changes of ICER according to different time-horizon scenarios. [file 1475-2875-13-66-S4.docx]

**Additional File 4 ICER by time horizon**

**Changes of ICER according to different time-horizon scenarios**

| Time-horizon | Societal perspective  (Including productivity) | | Health services perspective  (No productivity) | |
| --- | --- | --- | --- | --- |
|  | Strategy ranking | Incr C/E (ICER) | Strategy ranking | Incr C/E (ICER) |
| 1 year |  |  |  |  |
|  | LLINs | $306.94 | LLINs | $539.42 |
|  | Vaccines | (Dominated) | Vaccines | (Dominated) |
| 5 years | LLINs |  | No Intervention |  |
|  | No Intervention | (Dominated) | LLINs | $22.40 |
|  | Vaccines | (Dominated) | Vaccines | (Dominated) |
| 10 years | LLINs |  | No Intervention |  |
|  | No Intervention | (Dominated) | LLINs | $10.74 |
|  | Vaccines | $681.71 | Vaccines | $732.73 |
| 20 years | LLINs |  | No Intervention |  |
|  | Vaccines | $127.32 | LLINs | $5.95 |
|  | No Intervention | (Dominated) | Vaccines | $268.43 |
| 30 years | Vaccines |  | No Intervention |  |
|  | LLINs | (Dominated) | LLINs | $4.54 |
|  | No Intervention | (Dominated) | Vaccines | $186.13 |
| 40 years | Vaccines |  | No Intervention |  |
|  | LLINs | (Dominated) | LLINs | $3.94 |
|  | No Intervention | (Dominated) | Vaccines | $155.59 |
| 50 years | Vaccines |  | No Intervention |  |
|  | LLINs | (Dominated) | LLINs | $3.66 |
|  | No Intervention | (Dominated) | Vaccines | $142.08 |
| 60 years | Vaccines |  | No Intervention |  |
|  | LLINs | (Dominated) | LLINs | $3.52 |
|  | No Intervention | (Dominated) | Vaccines | $135.56 |
