# Supplementary material for: Cost-effectiveness analysis of vaccinating children in Malawi with RTS,S vaccines in comparison with long-lasting insecticide-treated nets
Source: Malar J. 2014 Feb 24;13:66. doi: 10.1186/1475-2875-13-66 (PMC4016032; doi:10.1186/1475-2875-13-66)
Supplement: Additional file 5: Table — Changes in vaccine’s ICER according to different discount rates. [file 1475-2875-13-66-S5.docx]

**Additional File 5 ICER by discount rates**

***Changes in vaccine’s ICER according to different discount rates***

| **Discount rate** | **Societal perspective** | | **Health services perspective** | |
| --- | --- | --- | --- | --- |
|  | **Strategy ranking** | **Incremental cost / DALY averted (ICER)** | **Strategy ranking** | **Incremental cost / DALY averted (ICER)** |
| 0% | Vaccines |  | No Intervention |  |
|  | LLINs  No Intervention | (Dominated)  (Dominated) | LLINs  Vaccines | $1.99  $73.69 |
| 3% | Vaccines |  | No Intervention |  |
|  | LLINs  No Intervention | (Dominated)  (Dominated) | LLINs  Vaccines | $3.72  $145.03 |
| 5% | Vaccines |  | No Intervention |  |
|  | LLINs  No Intervention | (Dominated)  (Dominated) | LLINs  Vaccines | $5.23  $213.53 |
| 6% | LLINs |  | No Intervention |  |
|  | Vaccines  No Intervention | $50.53  (Dominated) | LLINs  Vaccines | $6.08  $255.01 |
